# Bis(pyridinium) tetra­chlorido­zincate(II), [Hpy]_2_[ZnCl_4_]

**DOI:** 10.1107/S241431462601000X

**Published:** 2026-09-30

**Authors:** Hans Reuter

**Affiliations:** a Chemistry, Osnabrück University, Barbarastr. 7, 49069 Osnabrück, Germany; Howard University, USA

**Keywords:** crystal structure, hydrogen bonds, tetra­chlorido­zincate, π–π inter­actions

## Abstract

Single crystals of the title compound, (C_5_H_6_N)_2_[ZnCl_4_] or [Hpy]_2_[ZnCl_4_], were grown from an aqueous solution of pyridinium chloride and zinc dichloride. The compound is isostructural with the corresponding cobalt compound and exhibits an almost tetra­hedral [ZnCl_4_]^2−^ anion and two different pyridinium cations. The structure is described with respect to the bond lengths and angles of the anion and the π–π inter­actions between the cations, whilst mention is made of the N—H⋯Cl and C—H⋯Cl inter­actions already known from the cobalt compound.

## Abstract 2

The structure of the title compound is described in terms of the isostructural cobalt compound with particular regard on the different π–π inter­actions between the pyridinium ions.

## Graphical abstract

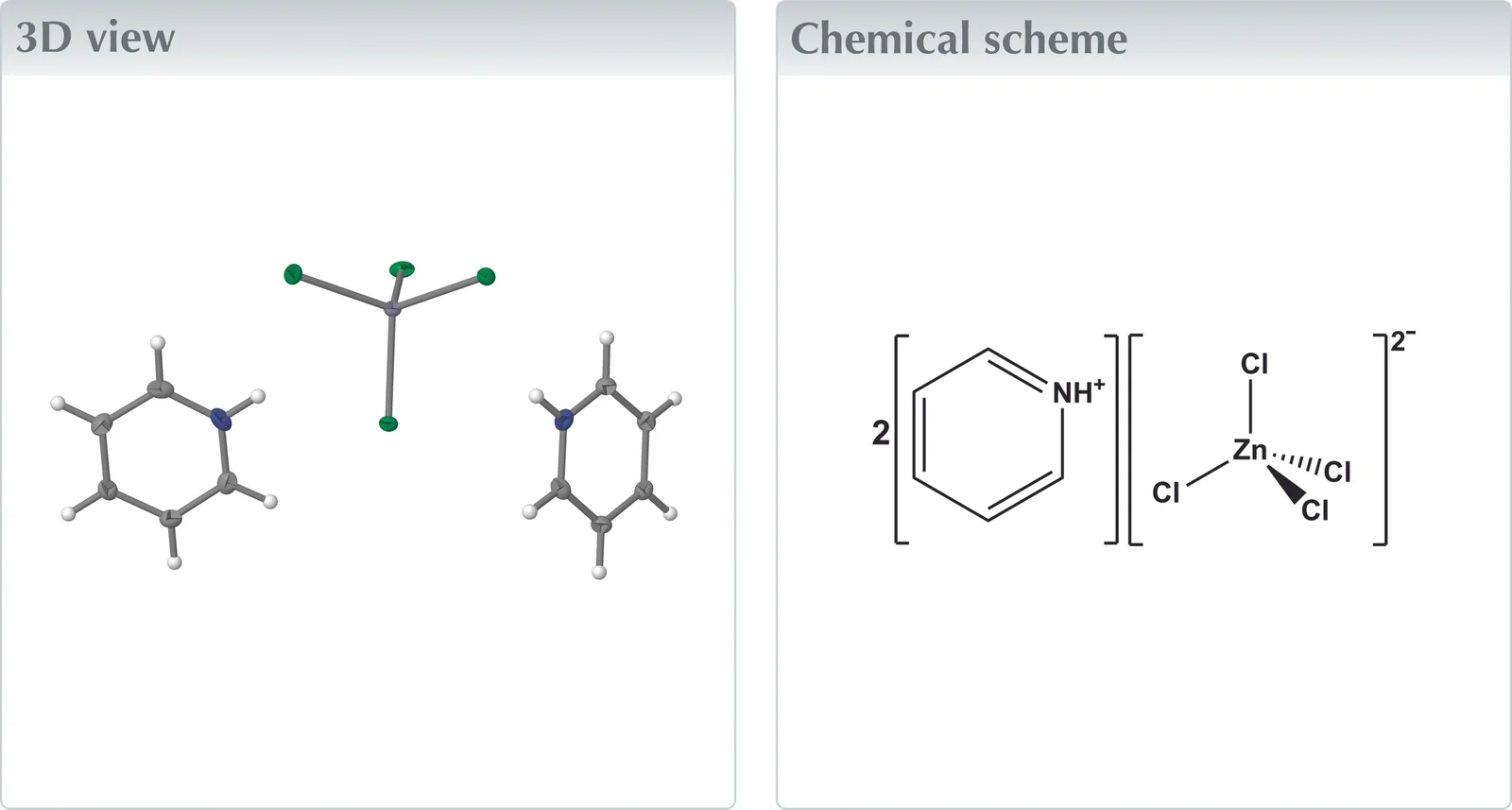

## Structure description

The title compound is isostructural with the analogous cobalt compound (Felloni *et al.*, 2004 ▸) and crystallizes in the triclinic space group *P*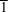 with one formula unit in the asymmetric unit (Fig. 1 ▸). The zinc atom is almost tetra­hedrally [〈(Cl—Zn—Cl) = 105.62 (1)–111.91 (1)°, 〈(Cl—Zn—Cl)_mean_ = 109.5 (23)°] coordinated from four chlorine atoms. The zinc–chlorine distances are, on average, slightly shorter [*d*(Zn—Cl)_mean_ = 2.273 (11) Å] than the cobalt-chlorine distances [*d*(Co—Cl)_mean_ = 2.276 (7) Å], but vary much more widely [2.2605 (3) − 2.2862 (3) Å] than the latter. Zn—Cl distances of similar length and variation can be found, for example, in the compound with the 4,4′-bipyridinium ion [*d*(Zn—Cl)_mean_ = 2.275 (27) Å] (Gillon *et al.*, 2000 ▸) and in the compound with the 3-chloro­pyridinium ion [*d*(Zn—Cl)_mean_ = 2.272 (14) Å] (Mínguez Espallargas *et al.*, 2009 ▸).

As the publication on the cobalt compound (Felloni *et al.*, 2004 ▸) describes the key aspects of the crystal structure, particularly with regard to the pattern of N—H⋯Cl hydrogen bonds and C—H⋯Cl contacts, there is no need to provide a detailed description of this here. All the necessary information to these supra­molecular inter­actions can be found in Table 1 ▸ and can be compared directly with the values reported for the cobalt compound, as the asymmetric unit has been adjusted in terms of atomic numbering and its position within the unit cell.

However, another aspect is presented here that is not given sufficient attention in the publication on the cobalt compound. Within the crystal structure (Fig. 2 ▸), the two pyridinium ions are arranged such that they partially overlap: Hpy(1) on one side, Hpy(2) on both sides. In all three cases, the relevant element of symmetry is a center of symmetry. In the case of Hpy(1), this is located at Wyckhoff position *e*; in the case of Hpy(2), at Wyckhoff positions *c* and *h*. The centers of symmetry ensure that the planes of the two neighboring pyridinium ions are exactly parallel to one another, with the nitro­gen atoms facing in opposite directions.

It can be seen from Fig. 3 ▸ that the more the pyridinium ions overlap, the greater the distance between the planes becomes. The different distances between the nitro­gen atoms in these arrangements is also evident.

## Synthesis and crystallization

The title compound was synthesized by dissolving equimolar qu­anti­ties of zinc chloride and pyridinium chloride in water. When the solvent evaporates, the compound crystallizes in transparent, slightly green needles.

## Refinement

Crystal data,data collection and structure refinement details are summarized in Table 2 ▸. Once it had become apparent that the compound was isostructural with the cobalt compound, its asymmetric unit was used to describe the structure.

## Supplementary Material

Crystal structure: contains datablock(s) I. DOI: 10.1107/S241431462601000X/bv4063sup1.cif

Structure factors: contains datablock(s) I. DOI: 10.1107/S241431462601000X/bv4063Isup2.hkl

CCDC reference: 2590726

Additional supporting information:  crystallographic information; 3D view; checkCIF report

## Figures and Tables

**Figure 1:**
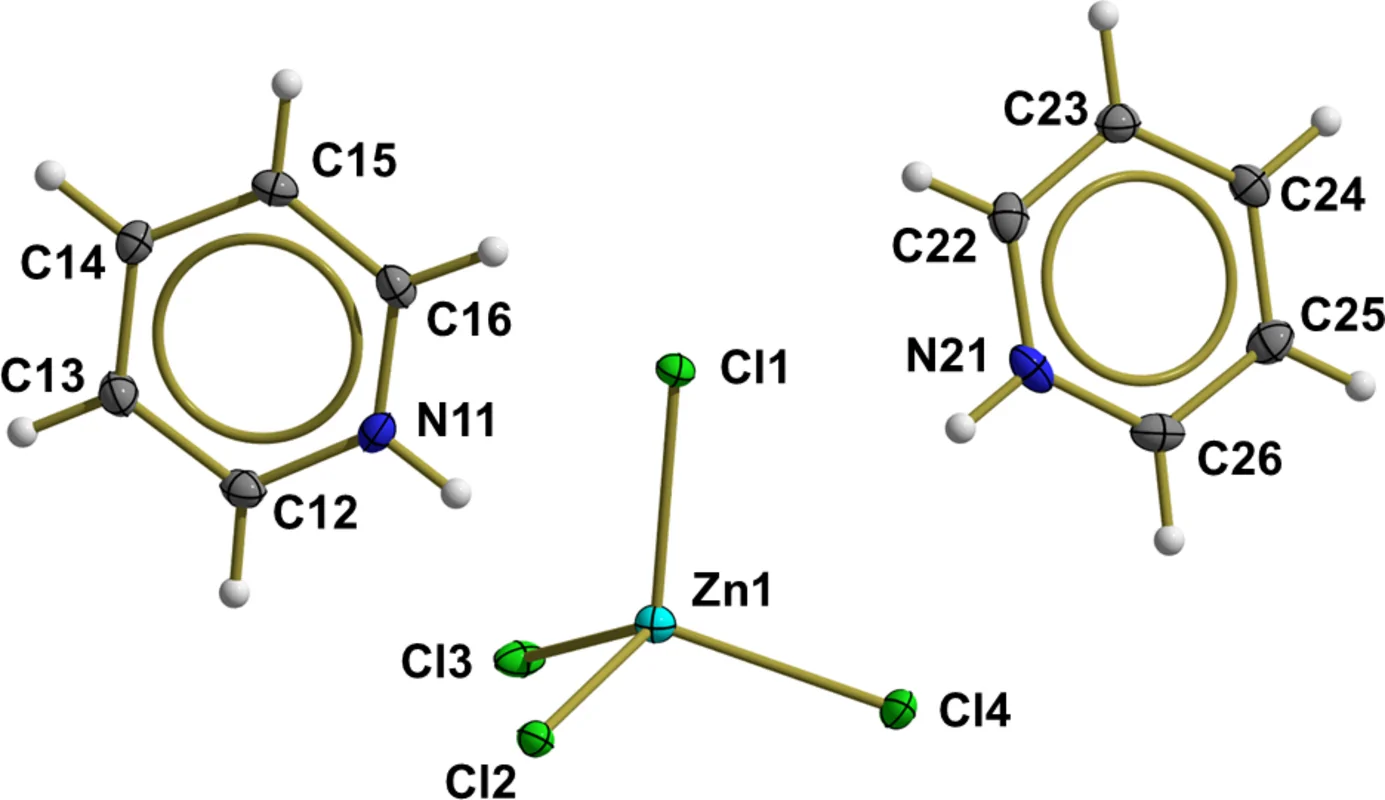
Asymmetric unit of the crystal structure of [Hpy]_2_[ZnCl_4_] with atom numbering according to the isostructural cobalt compound. With the exception of the hydrogen atoms, which are shown as spheres of arbitrary radius, all other atoms are drawn as anisotropic displacement ellipsoids at the 50% probability level.

**Figure 2:**
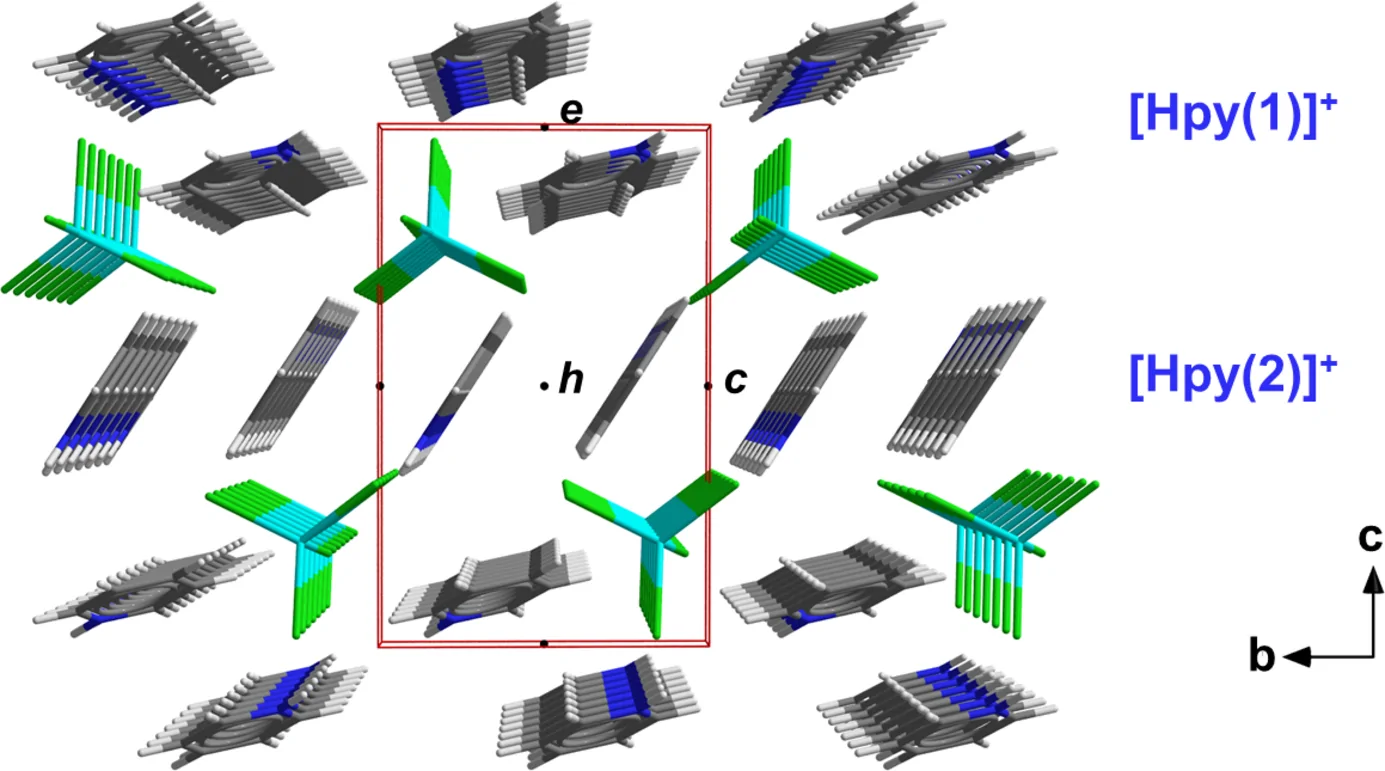
Ball-and-stick model of the ions in the crystal structure of the title compound showing their three-dimensional packing when looking down the *a* axis. The layers of the two different pyridinium ions are labeled and the relevant centers of symmetry (black dots) marked with Wyckhoff letters. Color code: Zn = light blue, Cl = yellow–green, N = dark blue, C = dark gray, H = white.

**Figure 3:**
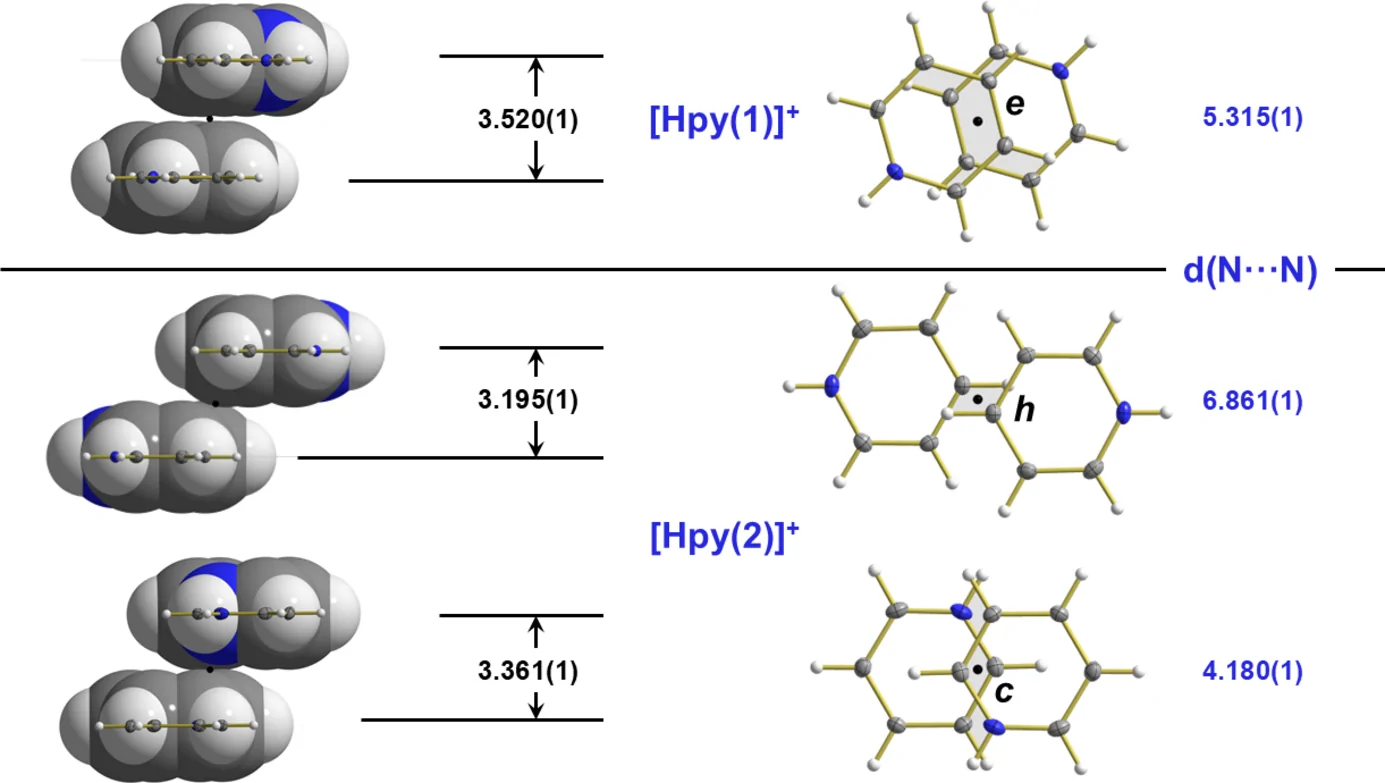
Different representations of the cation stacking. (left) Space-filling model with stick model overlayed; side-view with distances between the least-squares planes (Å, black values)*.* (right) Ball-and-stick models of the centrosymmetric dimers looking down on the least-square planes with distances (Å, blue values])between the nitro­gen atoms and positions of the relevant centers of symmetry (black dots) marked with the Wyckhoff letters.

**Table 1:** Hydrogen-bond geometry (Å, °)

*D*—H⋯*A*	*D*—H	H⋯*A*	*D*⋯*A*	*D*—H⋯*A*
N11—H11⋯Cl2	0.88	2.51	3.211 (1)	137
N11—H11⋯Cl2^i^	0.88	2.73	3.350 (1)	129
C12—H12⋯Cl2	0.95	2.97	3.443 (1)	112
C13—H13⋯Cl2^ii^	0.95	2.94	3.584 (1)	127
C15—H15⋯Cl1^iii^	0.95	2.96	3.576 (1)	124
C16—H16⋯Cl2^i^	0.95	2.84	3.417 (1)	120
N21—H21⋯Cl1	0.88	2.69	3.337 (1)	132
N21—H21⋯Cl4	0.88	2.53	3.246 (1)	139
C22—H22⋯Cl1	0.95	2.83	3.416 (1)	121
C22—H22⋯Cl4^iv^	0.95	2.97	3.592 (1)	125
C23—H23⋯Cl4^iv^	0.95	2.90	3.559 (1)	128
C25—H25⋯Cl3^v^	0.95	2.80	3.552 (1)	137
C26—H26⋯Cl4	0.95	2.96	3.466 (2)	115

Symmetry codes: (i) 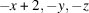; (ii) 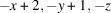; (iii) 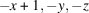; (iv) 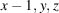; (v) 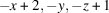.

**Table 2:** Experimental details

Crystal data	
Chemical formula	(C_5_H_6_N)_2_[ZnCl_4_]
*M*_r_	367.39
Crystal system, space group	Triclinic, *P* 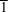
Temperature (K)	100
*a*, *b*, *c* (Å)	7.5711 (3), 7.9451 (3), 12.3798 (5)
α, β, γ (°)	88.942 (2), 83.518 (2), 79.959 (2)
*V* (Å^3^)	728.59 (5)
*Z*	2
Radiation type	Mo *K*α
μ (mm^−1^)	2.40
Crystal size (mm)	0.39 × 0.24 × 0.19

Data collection	
Diffractometer	Bruker APEXII CCD
Absorption correction	Multi-scan (*SADABS*; Krause *et al.*, 2015 ▸)
*T*_min_, *T*_max_	0.498, 0.523
No. of measured, independent and observed [*I* > 2σ(*I*)] reflections	82340, 4103, 3846
*R*_int_	0.030
(sin θ/λ)_max_ (Å^−1^)	0.696

Refinement	
*R*[*F*^2^ > 2σ(*F*^2^)], *wR*(*F*^2^), *S*	0.016, 0.041, 1.07
No. of reflections	4103
No. of parameters	156
H-atom treatment	Only H-atom displacement parameters refined
Δρ_max_, Δρ_min_ (e Å^−3^)	0.38, −0.28

Computer programs: *APEX2* and *SAINT* (Bruker, 2009 ▸), *SHELXS97* (Sheldrick, 2008 ▸), *SHELXL2014/7* (Sheldrick, 2015 ▸), *DIAMOND* (Brandenburg, 2006 ▸), *Mercury* (Macrae *et al.*, 2020 ▸) and *publCIF* (Westrip, 2010 ▸).

## full crystallographic data

### Bis(pyridinium) tetrachloridozincate(II) Crystal data

**Table:**

(C_5_H_6_N)_2_[ZnCl_4_]	*Z* = 2
*M**_r_* = 367.39	*F*(000) = 368
Triclinic, *P*1	*D*_x_ = 1.675 Mg m^−^^3^
*a* = 7.5711 (3) Å	Mo *K*α radiation, λ = 0.71073 Å
*b* = 7.9451 (3) Å	Cell parameters from 9982 reflections
*c* = 12.3798 (5) Å	θ = 2.6–29.6°
α = 88.942 (2)°	µ = 2.40 mm^−^^1^
β = 83.518 (2)°	*T* = 100 K
γ = 79.959 (2)°	Needle, green
*V* = 728.59 (5) Å^3^	0.39 × 0.24 × 0.19 mm

### Bis(pyridinium) tetrachloridozincate(II) Data collection

**Table:**

Bruker APEXII CCD diffractometer	3846 reflections with *I* > 2σ(*I*)
φ and ω scans	*R*_int_ = 0.030
Absorption correction: multi-scan (SADABS; Krause *et al.*, 2015)	θ_max_ = 29.6°, θ_min_ = 2.6°
*T*_min_ = 0.498, *T*_max_ = 0.523	*h* = −10→10
82340 measured reflections	*k* = −11→11
4103 independent reflections	*l* = −17→17

### Bis(pyridinium) tetrachloridozincate(II) Refinement

**Table:**

Refinement on *F*^2^	Primary atom site location: structure-invariant direct methods
Least-squares matrix: full	Hydrogen site location: inferred from neighbouring sites
*R*[*F*^2^ > 2σ(*F*^2^)] = 0.016	Only H-atom displacement parameters refined
*wR*(*F*^2^) = 0.041	*w* = 1/[σ^2^(*F*_o_^2^) + (0.0205*P*)^2^ + 0.2351*P*] where *P* = (*F*_o_^2^ + 2*F*_c_^2^)/3
*S* = 1.07	(Δ/σ)_max_ = 0.002
4103 reflections	Δρ_max_ = 0.38 e Å^−^^3^
156 parameters	Δρ_min_ = −0.28 e Å^−^^3^
0 restraints	

### Bis(pyridinium) tetrachloridozincate(II) Special details

**Table:**

Geometry. All esds (except the esd in the dihedral angle between two l.s. planes) are estimated using the full covariance matrix. The cell esds are taken into account individually in the estimation of esds in distances, angles and torsion angles; correlations between esds in cell parameters are only used when they are defined by crystal symmetry. An approximate (isotropic) treatment of cell esds is used for estimating esds involving l.s. planes.
Refinement. The H atoms were refined in idealized geometries and allowed to ride on the parent carbon [*d*(C—H) = 0.95 Å] and nitrogen atoms [*d*(N—H) = 0.88 Å] with one common isotropic temperature factor for each of the two pyridinium ions.

### Bis(pyridinium) tetrachloridozincate(II) Fractional atomic coordinates and isotropic or equivalent isotropic displacement parameters (Å^2^)

**Table:**

	*x*	*y*	*z*	*U*_iso_*/*U*_eq_	
Zn1	0.94436 (2)	0.17307 (2)	0.22056 (2)	0.01280 (4)	
Cl1	0.67207 (3)	0.09965 (3)	0.19775 (2)	0.01488 (5)	
Cl2	1.10183 (3)	0.18295 (3)	0.05356 (2)	0.01455 (5)	
Cl3	0.90410 (3)	0.43146 (3)	0.30190 (2)	0.01870 (6)	
Cl4	1.08368 (3)	−0.03785 (3)	0.32370 (2)	0.01898 (6)	
N11	0.75046 (12)	0.23776 (12)	−0.07461 (8)	0.01706 (18)	
H11	0.8367	0.1659	−0.0479	0.0247 (15)*	
C12	0.77721 (15)	0.39734 (15)	−0.09665 (9)	0.0185 (2)	
H12	0.8877	0.4310	−0.0845	0.0247 (15)*	
C13	0.64300 (15)	0.51229 (14)	−0.13719 (9)	0.0185 (2)	
H13	0.6597	0.6262	−0.1534	0.0247 (15)*	
C14	0.48320 (15)	0.45976 (14)	−0.15402 (9)	0.0170 (2)	
H14	0.3889	0.5381	−0.1812	0.0247 (15)*	
C15	0.46079 (14)	0.29270 (14)	−0.13117 (9)	0.0170 (2)	
H15	0.3518	0.2555	−0.1429	0.0247 (15)*	
C16	0.59836 (15)	0.18200 (14)	−0.09133 (9)	0.0181 (2)	
H16	0.5860	0.0668	−0.0757	0.0247 (15)*	
N21	0.70751 (13)	−0.16465 (12)	0.40981 (8)	0.01933 (19)	
H21	0.7710	−0.1133	0.3597	0.0294 (17)*	
C22	0.53087 (16)	−0.15652 (14)	0.40250 (9)	0.0194 (2)	
H22	0.4764	−0.0965	0.3443	0.0294 (17)*	
C23	0.42975 (15)	−0.23567 (14)	0.47987 (9)	0.0188 (2)	
H23	0.3045	−0.2314	0.4757	0.0294 (17)*	
C24	0.51212 (15)	−0.32184 (14)	0.56405 (9)	0.0181 (2)	
H24	0.4433	−0.3767	0.6183	0.0294 (17)*	
C25	0.69495 (16)	−0.32800 (15)	0.56911 (9)	0.0210 (2)	
H25	0.7528	−0.3871	0.6265	0.0294 (17)*	
C26	0.79136 (15)	−0.24706 (15)	0.48961 (10)	0.0211 (2)	
H26	0.9169	−0.2498	0.4915	0.0294 (17)*	

### Bis(pyridinium) tetrachloridozincate(II) Atomic displacement parameters (Å^2^)

**Table:**

	*U*^11^	*U*^22^	*U*^33^	*U*^12^	*U*^13^	*U*^23^
Zn1	0.01176 (6)	0.01293 (6)	0.01405 (6)	−0.00272 (4)	−0.00215 (4)	0.00042 (4)
Cl1	0.01383 (11)	0.01502 (11)	0.01738 (11)	−0.00575 (8)	−0.00378 (9)	0.00149 (8)
Cl2	0.01337 (11)	0.01618 (11)	0.01457 (11)	−0.00401 (8)	−0.00148 (8)	0.00135 (8)
Cl3	0.01431 (11)	0.01759 (12)	0.02475 (13)	−0.00271 (9)	−0.00354 (9)	−0.00729 (10)
Cl4	0.01502 (11)	0.02239 (13)	0.01953 (12)	−0.00225 (9)	−0.00462 (9)	0.00743 (10)
N11	0.0159 (4)	0.0177 (4)	0.0164 (4)	0.0011 (3)	−0.0035 (3)	0.0023 (3)
C12	0.0170 (5)	0.0223 (5)	0.0180 (5)	−0.0073 (4)	−0.0037 (4)	0.0005 (4)
C13	0.0224 (5)	0.0154 (5)	0.0194 (5)	−0.0064 (4)	−0.0049 (4)	0.0030 (4)
C14	0.0173 (5)	0.0170 (5)	0.0165 (5)	−0.0013 (4)	−0.0042 (4)	0.0013 (4)
C15	0.0154 (5)	0.0191 (5)	0.0174 (5)	−0.0059 (4)	−0.0014 (4)	−0.0018 (4)
C16	0.0205 (5)	0.0141 (5)	0.0195 (5)	−0.0047 (4)	0.0006 (4)	0.0010 (4)
N21	0.0232 (5)	0.0171 (4)	0.0178 (4)	−0.0080 (4)	0.0051 (4)	−0.0022 (3)
C22	0.0252 (6)	0.0161 (5)	0.0174 (5)	−0.0035 (4)	−0.0043 (4)	0.0000 (4)
C23	0.0155 (5)	0.0189 (5)	0.0226 (5)	−0.0039 (4)	−0.0036 (4)	−0.0007 (4)
C24	0.0195 (5)	0.0186 (5)	0.0163 (5)	−0.0052 (4)	0.0007 (4)	−0.0002 (4)
C25	0.0207 (5)	0.0246 (6)	0.0180 (5)	−0.0024 (4)	−0.0054 (4)	0.0000 (4)
C26	0.0146 (5)	0.0231 (6)	0.0259 (6)	−0.0047 (4)	−0.0007 (4)	−0.0061 (4)

### Bis(pyridinium) tetrachloridozincate(II) Geometric parameters (Å, º)

**Table:**

Zn1—Cl3	2.2605 (3)	C15—H15	0.9500
Zn1—Cl4	2.2715 (3)	C16—H16	0.9500
Zn1—Cl2	2.2742 (3)	N21—C26	1.3344 (16)
Zn1—Cl1	2.2862 (3)	N21—C22	1.3408 (15)
N11—C12	1.3369 (15)	N21—H21	0.8800
N11—C16	1.3418 (14)	C22—C23	1.3708 (16)
N11—H11	0.8800	C22—H22	0.9500
C12—C13	1.3759 (16)	C23—C24	1.3840 (16)
C12—H12	0.9500	C23—H23	0.9500
C13—C14	1.3847 (15)	C24—C25	1.3850 (16)
C13—H13	0.9500	C24—H24	0.9500
C14—C15	1.3873 (15)	C25—C26	1.3759 (17)
C14—H14	0.9500	C25—H25	0.9500
C15—C16	1.3719 (15)	C26—H26	0.9500
			
Cl3—Zn1—Cl4	111.911 (12)	N11—C16—C15	119.44 (10)
Cl3—Zn1—Cl2	109.683 (11)	N11—C16—H16	120.3
Cl4—Zn1—Cl2	111.059 (11)	C15—C16—H16	120.3
Cl3—Zn1—Cl1	110.461 (10)	C26—N21—C22	122.99 (10)
Cl4—Zn1—Cl1	105.621 (11)	C26—N21—H21	118.5
Cl2—Zn1—Cl1	107.971 (10)	C22—N21—H21	118.5
C12—N11—C16	123.13 (10)	N21—C22—C23	119.32 (10)
C12—N11—H11	118.4	N21—C22—H22	120.3
C16—N11—H11	118.4	C23—C22—H22	120.3
N11—C12—C13	119.30 (10)	C22—C23—C24	119.27 (10)
N11—C12—H12	120.4	C22—C23—H23	120.4
C13—C12—H12	120.4	C24—C23—H23	120.4
C12—C13—C14	119.11 (10)	C23—C24—C25	119.95 (10)
C12—C13—H13	120.4	C23—C24—H24	120.0
C14—C13—H13	120.4	C25—C24—H24	120.0
C13—C14—C15	120.01 (10)	C26—C25—C24	118.84 (11)
C13—C14—H14	120.0	C26—C25—H25	120.6
C15—C14—H14	120.0	C24—C25—H25	120.6
C16—C15—C14	118.99 (10)	N21—C26—C25	119.63 (10)
C16—C15—H15	120.5	N21—C26—H26	120.2
C14—C15—H15	120.5	C25—C26—H26	120.2

### Bis(pyridinium) tetrachloridozincate(II) Hydrogen-bond geometry (Å, º)

**Table:**

*D*—H···*A*	*D*—H	H···*A*	*D*···*A*	*D*—H···*A*
N11—H11···Cl2	0.88	2.51	3.211 (1)	137
N11—H11···Cl2^i^	0.88	2.73	3.350 (1)	129
C12—H12···Cl2	0.95	2.97	3.443 (1)	112
C13—H13···Cl2^ii^	0.95	2.94	3.584 (1)	127
C15—H15···Cl1^iii^	0.95	2.96	3.576 (1)	124
C16—H16···Cl2^i^	0.95	2.84	3.417 (1)	120
N21—H21···Cl1	0.88	2.69	3.337 (1)	132
N21—H21···Cl4	0.88	2.53	3.246 (1)	139
C22—H22···Cl1	0.95	2.83	3.416 (1)	121
C22—H22···Cl4^iv^	0.95	2.97	3.592 (1)	125
C23—H23···Cl4^iv^	0.95	2.90	3.559 (1)	128
C25—H25···Cl3^v^	0.95	2.80	3.552 (1)	137
C26—H26···Cl4	0.95	2.96	3.466 (2)	115

Symmetry codes: (i) −*x*+2, −*y*, −*z*; (ii) −*x*+2, −*y*+1, −*z*; (iii) −*x*+1, −*y*, −*z*; (iv) *x*−1, *y*, *z*; (v) −*x*+2, −*y*, −*z*+1.
